# Adherence and persistence rates of major antidiabetic medications: a review

**DOI:** 10.1186/s13098-022-00785-1

**Published:** 2022-01-15

**Authors:** David Seung U. Lee, Howard Lee

**Affiliations:** 1 Department of Molecular Medicine and Biopharmaceutical Sciences, Graduate School of Convergence Science and Technology, Seoul National University, Seoul, 08826 South Korea; 2 Department of Applied Biomedical Engineering, Graduate School of Convergence Science and Technology, Seoul National University, Seoul, 08826 South Korea; 3 Department of Clinical Pharmacology and Therapeutics, Seoul National University College of Medicine and Hospital, 103 Daehak-ro, Jongno-gu, Seoul, 110-799 Republic of Korea; 4 Center for Convergence Approaches in Drug Development, Graduate School of Convergence Science and Technology, Seoul National University, Seoul, 08826 South Korea; 5 Advanced Institute of Convergence Technology, Suwon, 16229 South Korea

**Keywords:** Diabetes mellitus, Adherence, Persistence, Antidiabetic medications, Hypoglycemic agents

## Abstract

The objective of this paper was to review the adherence and persistence rates of major antidiabetic medication classes (i.e., metformin, sulfonylureas, sodium glucose cotransporter-2 inhibitors, dipeptidyl peptidase-4 inhibitors, insulin, glucagon-like peptide-1 receptor agonists, and thiazolidinediones) by summarizing the major findings of the studies published since 2017. In addition, we reported the potential causes for low adherence and persistence of antidiabetic medications. Based on the literature, the highest rate of adherence and persistence was consistently observed in metformin users. Second to metformin were sodium glucose cotransporter-2 inhibitors. Injectable therapies such as insulin and glucagon-like peptide-1 receptor agonists trailed low on the adherence and persistence rates. To the best of our knowledge, no studies published since the year 2017 analyzed the adherence and persistence of thiazolidinediones independently. The most frequently cited cause for low adherence and persistence was the severity of adverse events. Baseline characteristics (e.g., baseline HbA1c level), demographic information (e.g., age, gender, or ethnicity), and comorbidity profiles also had significant impacts on adherence and persistence in patients with type 2 diabetes mellitus.

## Background

Adequate management of chronic disease is difficult. Patients are often required to take one or more medications over the entire lifespan of the disease [1]. Management of chronic disease is further complicated by two patterns of medication non-use: (1) missed medication doses (termed non-adherence in this study) and (2) abrupt discontinuation or substantial medication gap (termed non-persistence or discontinuation in this study) [2]. In developed countries, average adherence to medications for chronic diseases is as low as 50%, while the measure is lower in developing countries due to limited access to healthcare resources [3, 4]. Medication non-use aggravates the burden of chronic diseases and clinical outcomes of patients [4, 5]. Therefore, ensuring adherence and persistence of medications is key to successful management of chronic disease.

Poor adherence and persistence remain a barrier to optimal care for patients with type 2 diabetes mellitus (T2DM) [6–9]. A systematic review found that only 56.2% in T2DM patients continued treatment one year after treatment initiation [10]. Adherence and persistence to injection drugs are even lower. The persistence rate of insulin glargine in the first year after initiation is below 50% [11]. Suboptimal persistence undermines clinical outcomes, leading to poor glycemic control [12, 13] and increases mortality and comorbidity burden [14, 15]. Moreover, low adherence to antidiabetic medications increases healthcare costs and diminishes quality of life [5, 14, 16].

The causes of low adherence and persistence to T2DM medications are multifactorial [17]. The World Health Organization classified reasons for medication non-use into five categories: patient-related (e.g., age), socioeconomic (e.g., medication costs), condition-related (e.g., presence of complications), health-system-related (e.g., level of continuity of care), and medication-related (e.g., adverse effects) [4]. Similarly, motivations behind medication non-use in T2DM patients on injection therapies are multifaceted. Ineffective communication between patients and providers, inadequate knowledge about medications, and confusing directions for medication use simultaneously undermine treatment processes [18]. Moreover, the classes of antidiabetic medication influence the adherence and persistence to the treatment [1, 19].

The objective of this paper was to review the latest adherence and persistence rates of major antidiabetic medication classes chosen based on their proportional market shares [20]. Moreover, we compared the adherence and persistence rates of individual antidiabetic medications within the same medication class. Moreover, we investigated the potential causes for low adherence and persistence of antidiabetic medications. To this end, we summarized the major findings of the studies published since 2017. The year 2017 was chosen to account for the shift in pharmacological diabetes treatment pattern, as reflected in the medications’ proportional market shares [20], due in large part to the accelerating acceptance and widespread use of such medication classes as sodium glucose co-transporter 2 inhibitors and glucagon-like peptide-1 receptor agonists. Published studies on the topic of adherence and persistence of the selected antidiabetic medications were identified by searching the four databases, i.e., PubMed, Cochrane Library, Google Scholar, and Embase. This information will help guide clinical decisions to optimize treatment adherence and persistence in patients with T2DM while reducing complications and healthcare costs. A precise understanding about the causes of medication non-use will also assist the development of new antidiabetic drugs and delivery devices better equipped to improve adherence and persistence. The key findings of previous studies that analyzed the adherence and persistence of various antidiabetic medications are summarized in Table 1.

**Table 1:** Summary of previous studies on adherence and persistence of major antidiabetic medications

Study	Data source	Adherence measure	Persistence measure	Study population	Follow-up period	Key findings
*Metformin*						
Horsburgh et al., 2021 [26]	The Ministry of Health’s Virtual Diabetes Register (VDR)	N.A	Having a gap of ≥ 90 days	85,066 T2DM patients aged 18 or older in New Zealand who initiated monotherapy between January 1st 2006 and September 30^th^ 2014	1 year to 5 year	At year 1 after cohort entry, 28.2% of cohort members had discontinued metformin monotherapy at least once and 62.8% remained persistent; at year 2, 36.8% had discontinued at least once and 48.5% remained persistent; at year 5, 46.3% discontinued at least once and 27.7% remained persistent
Flory et al., 2017 [7]	The OptumLabs Data Warehouse (OLDW)	Daily medication possession probability (daily MPP)	N.A	11,067 T2DM patients aged 18 or older (37.2% metformin users) who received a first electronic prescription for one of the index medications during the 2012 calendar year	1 year	The daily MPP of metformin-prescribed patients during one year of follow-up was 0.46
Flory et al., 2018 [28]	The Weill Cornell Medicine Database	N.A	Absence of any further metformin prescriptions after the first 90 days	1,259 treatment-naïve T2DM patients who initiated metformin therapy between January 1st 2009 and September 31st 2015	> 1.5 year	The overall rate of early discontinuation was 20.3%
Naffaa et al., 2020 [84]	Maccabi Healthcare Services	Proportion of days covered (PDC)	N.A	113,749 T2DM patients aged 18 or older in Israel who initiated metformin between 1998 and 2014	1 year	30.8% of patients demonstrated adherence (PDC ≥ 0.8)
Walker et al., 2020 [85]	Diabetes Prevention Program Outcomes Study (DPPOS) Follow-up Data	Percentage of pill taken	N.A	664 T2DM patients who were enrolled in Diabetes Prevention Program (DPP) and had taken adherence assessment semiannually	11 years	Overall the cumulative adherence in 11 years was 60%
Nishimura et al., 2019 [27]	The Japan Medical Data Center (JMDC) database and the Medical Data Vision (MDV) database	Proportion of days covered (PDC)	Percentage of patients who remained on treatment after one-year follow-up	40,908 and 90,421 (from JMDC and MDV, respectively) adult T2DM patients aged 18 or older in Japan during January 2011 to December 2015	1 year	Twelve-month persistence to metformin was 57.3% and 73.8% for treatment naïve patients identified from JMDC and MDV, respectively; twelve-month persistence was 69.3% and 73.6% for previously treated patients identified from JMDC and MDV; the corresponding twelve-month adherence was 0.86, 0.97, 0.83, and 0.95
*Sulfonylureas*						
Bell et al., 2017 [31]	The Truven Health Market-Scan®; Commercial Claims and Encounters; Medicare Supplemental and Coordination of Benefits; Early View databases	Proportion of days covered (PDC)	The number of days from the index date until the earlier of a discontinuation of the index medication class or the end of follow-up	25,490 T2DM patients (aged 18 and above) with at least 1 outpatient pharmacy claims for sulfonylurea between January 1, 2015 and December 31, 2015	6 months	The average PDC was 0.72; the proportion of adherent patients (with PDC ≥ 0.8) was 53.9%; 31.1% of the patients discontinued (not persistent)
Popoviciu et al., 2019 [38]	Clinical charts, laboratory parameters reviews, and cross-sectional survey data in Romania	Patient-report questionnaire	N.A	385 T2DM patients (30 years of age or older) who have been taking sulfonylureas (monotherapy or in combination therapy with metformin) for at least 6 months prior to enrollment	2 months	77% of patients adhered to their prescriptions every day
Bloomgarden et al., 2017 [32]	MarketScan® Commercial Claims and Encounters; Medicare Supplemental databases; Truven Health Analytics	Proportion of days covered (PDC)	The proportion of patients who continued to use their index medications	34,113 T2DM patients aged 18 years or older who initiated either sitagliptin or sulfonylurea as an add-on to metformin from January 1, 2009 to December 31, 2012	1 to 3 years	55.9% of patients on metformin and sulfonylurea were adherent (PDC ≥ 0.8) at Year 1; 49.9% remained adherent at Year 2; 47.1% remained adherent at Year 3; the adherence of patients on metformin and sitagliptin was higher than that of patients on metformin and sulfonylurea (p < 0.001); the median time of discontinuation for patients on metformin and sitagliptin was 133 days longer
Carls et al., 2017 [36]	The Optum/Humedica SmartFile Database	Proportion of days covered (PDC)	Absence of a 30-day gap in medications on hand without subsequent fills	5,818 T2DM patients aged 18 years or older who initiated the index medication between January 2007 and December 2014 (2,713 sulfonylurea users)	1 year	The average PDC was 0.62; 40% of patients showed good adherence (PDC ≥ 0.8); 38% of patients discontinued before the end of follow-up
McGovern et al., 2018 [1]	The Royal College of General Practitioners Research and Surveillance Centre (RCGP-RSC) database	N.A	Absence of a gap in prescriptions of greater than or equal to 90 days; the duration of persistence was defined as the time interval between the first prescription and the last identified prescription	60,327 T2DM patients who initiated treatment with one of the index medications between January 1, 2005 and December 31, 2015 (20,819 sulfonylurea users)	6 months, 1 year, 2 years, and 5 years	The median duration of persistence of patients treated with sulfonylureas was 2.12 years; 76.6% of patients remained persistent at 6 months; at 1 year, 64.8% remained persistent; at 2 years, 51.3% remained persistent; at 5 years, 31.6% remained persistent; the hazard ratio (HR) of discontinuation of sulfonylureas to that of metformin via Cox regression analysis was 1.2 (95% CI 1.16 to 1.24, p < 0.001)
Nishimura et al., 2019 [27]	The Japan Medical Data Center (JMDC) database and the Medical Data Vision (MDV) database	Proportion of days covered (PDC)	Percentage of patients who remained on treatment after one-year follow-up	40,908 and 90,421 (from JMDC and MDV, respectively) adult T2DM patients aged 18 or older in Japan who had been issued at least one prescription for the index medication during January 2011 to December 2015	1 year	Twelve-month persistence rates were 50.4% and 56.0% for treatment naïve patients identified from JMDC and MDV, respectively; twelve-month persistence rates were 58.1% and 62.2% for previously treated patients identified from JMDC and MDV; twelve-month adherence was 0.86 and 0.96 for treatment naive patients identified from JMDC and MDV; twelve-month adherence was 0.85 and 0.97 for previously treated patients identified from JMDC and MDV respectively
*SGLT2 inhibitors*						
Cai et al., 2017 [41]	The QuintilesIMS PharMetrics Plus Health Plan Claims Database	Proportion of days covered (PDC); medication possession ratio (MPR)	The number of consecutive days until discontinuation or end of follow-up (discontinuation was defined as having ≥ 90 days of prescription gap)	23,720 T2DM patients aged 18 or older with at least 1 pharmacy claims for an index medication between February 1, 2014 and June 30, 2014 (6,546 canagliflozin users, 3,087 dapagliflozin users)	1 year	Mean PDC for canagliflozin was 0.71, and the proportion of adherent patients (PDC ≥ 0.8) were 56.2%; mean persistence for canagliflozin was 278.6 days; the proportion of patients who remained on canagliflozin therapy was 67.6%; mean PDC for dapagliflozin was 0.64, and the proportion of adherent patients (PDC ≥ 0.8) was 41.8%; mean persistence for dapagliflozin was 260.3 days; the proportion of patients who remained on dapagliflozin therapy was 57.4%
Fadini et al., 2019 [49]	The DARWIN (DApagliflozin Real World evidence)-T2D study	N.A	The presence of prescription for the index medication at the first available visit 3–12 months after treatment initiation	12,782 T2DM patients (aged 18 to 80) who have initiated dapagliflozin 10 mg as add-on to metformin and/or insulin from March 13, 2015 to December 31st, 2016 (1,701 dapagliflozin users)	1 year	48.9% of patients remained persistent; adjusted for covariates, the relative risk of discontinuation associated with dapagliflozin versus other antidiabetic medications was 1.32 (95% CI 1.17- 1.47, p < 0.001)
Thayer et al., 2017 [45]	The Optum Research Database	N.A	The number of days without a prescription gap of ≥ 90 days	2944 T2DM patients aged 18 years or older with at least 1 pharmacy claim for canagliflozin or sitagliptin between April 1, 2013 and December 31, 2013	9 months	29% of patients on canagliflozin discontinued while 41.5% of patients on sitagliptin discontinued (p < 0.001); the average days of persistent treatment with canagliflozin was longer than the average days of persistent treatment with sitagliptin (152 days vs. 139 days; p < 0.001)
McGovern et al., 2018 [2]	The Royal College of General Practitioners Research and Surveillance Centre (RCGP-RSC) database	N.A	Absence of a gap in prescriptions of greater than or equal to 90 days the duration of persistence was defined as the time interval between the first prescription and the last identified prescription	60,327 T2DM patients who initiated treatment with one of the index medications between January 1, 2005 and December 31, 2015 (1642 SGLT2 inhibitor users)	6 months, 1 year, 2 years, and 5 years	79.5% of patients remained persistent at 6 months; at 1 year, 69.5% remained persistent; at 2 years, 54.8% remained persistent; the hazard ratio (HR) of discontinuation of SGLT2 inhibitors to that of metformin via Cox regression analysis was 1.04 (95% CI 0.93–1.17, p = 0.458)
Tumminia et al., 2020 [48]	Electronic chart records from Diabetes Center at the Garibaldi Hospital (Catania, Italy)	N.A	Discontinuation rate (i.e., the proportion of patients who didn't continue treatment until the end of follow-up)	364 T2DM patients aged 65 years or older, who started treatment with SGLT2 inhibitor from June 2015 to June 2018	2 years	Discontinuation rate in patients aged between 65 and 70 was 34.2%; discontinuation rate in patients aged 70 or older was 36.1%; there was no significant group difference in discontinuation rate (p = 0.26)
Ito et al., 2019 [43]	The Japan Medical Data Center (JMDC) database and the Medical Data Vision (MDV) database	N.A	Percentage of patients who remained on treatment after one-year follow-up	1,641 T2DM patients aged 18 or older with at least 1 prescription record of SGLT2 inhibitor between April 1, 2014 and March 31, 2016	1 year	44.3% of patients from JMDC were persistent; 53.3% of patients from MDV were persistent
Ofori-Asenso et al., 2019 [42]	The National Pharmaceutical Benefits Scheme of Australia	Proportion of days covered (PDC)	Continuous use of SGLT2 inhibitors without a prescription gap of ≥ 90 days	11,981 T2DM patients (aged 18 and above) who newly initiated treatment with dapagliflozin or empagliflozin from September 2015 to August 2017 (5993 dapagliflozin users, 5988 empagliflozin users)	1 year	Mean PDC for both SGLT2 inhibitors was 0.79; 65.8% of patients treated with SGLT2 inhibitors were adherent (PDC ≥ 0.8); 72.1% (8644/11981) were persistent at 1 year; the use of empagliflozin was associated with being adherent and persistent than the use of dapagliflozin (OR 1.39, 95% CI 1.29 to 1.51 and OR 1.14, 95% CI 1.06–1.22, respectively)
Nishimura et al., 2019 [27]	The Japan Medical Data Center (JMDC) database and the Medical Data Vision (MDV) database	Proportion of days covered (PDC)	Percentage of patients who remained on treatment after one-year follow-up	40,908 and 90,421 (from JMDC and MDV, respectively) adult T2DM patients aged 18 or older in Japan who had been issued at least one prescription for the index medication during January 2011 to December 2015	1 year	Twelve-month persistence rates were 53.5% and 63.4% for treatment naïve patients identified from JMDC and MDV, respectively; twelve-month persistence rates were 62.8% and 66.4% for previously treated patients identified from JMDC and MDV; twelve-month adherence was 0.75 and 0.95 for treatment naive patients identified from JMDC and MDV; twelve-month adherence was 0.77 and 0.92 for previously treated patients identified from JMDC and MDV respectively
*DPP4 inhibitors*						
Rascati et al., 2017 [56]	Humana's administrative claims database	Proportion of days covered (PDC)	The number of days on an index medication before a gap in therapy of greater than 31 days	26,089 T2DM patients aged 18 or older with a prescription claim between July 1, 2011 and March 31, 2013, who were enrolled in a Medicare Advantage Prescription Drug (MAPD) plan or a commercial insurance plan	1 year	MAPD patients on sitagliptin, saxagliptin, and linagliptin showed mean one-year PDC of 0.72, 0.72, and 0.67, respectively; the percentages of adherent patients (PDC ≥ 0.8) were 49.9%, 50.9%, and 41.4% for sitagliptin, saxagliptin, and linagliptin; the percentages of discontinuation patients for sitagliptin, saxagliptin, and linagliptin were 66.5%, 64.7%, and 72.6%
Oh et al., 2019 [59]	The Medical Data Vision (MDV) database	Proportion of days covered (PDC)	The percentage of patients who continued treatment over one-year period	39,826 T2DM patients (aged 18 or older with a prescription claim between May 2015 and May 2018; 15,435 patients were treatment naïve, while 24,391 patients had previous treatment histories	1 year	Regardless of treatment history, adherence and persistence to the once-daily regimen was slightly higher than that of twice-daily regimen (p = 0.1187), while significantly higher than that of once-weekly regimen (p < 0.0001)
Moura et al., 2018 [86]	The MarketScan® commercial claims and encounters database	N.A	The number of days without a prescription gap of ≥ 90 days	54,318 T2DM patients aged 18 years or older newly dispensed with either NPH insulin or a DPP4 inhibitor as an add-on to metformin and sulfonylurea between January 2011 and December 2014 (50,338 DPP4 users)	1 year	Cox regression analysis results showed that HR of discontinuation for NPH insulin was 1.33 compared with DPP4 inhibitors (95% CI 1.27–1.40, p < 0.05), when adjusted for baseline patient characteristics
McGovern et al., 2018 [2]	The Royal College of General Practitioners Research and Surveillance Centre (RCGP-RSC) database	N.A	Absence of a gap in prescriptions of ≥ 90 days	60,327 T2DM patients who initiated treatment with one of the index medications between January 1, 2005 and December 31, 2015 (9614 DPP4 inhibitor users)	6 months, 1 year, 2 years, and 5 years	Median persistence was 1.69 years; percentages of persistent patients at 6 months, 1 year, 2 years, and 5 years were 76.1%, 62.2%, 45.5%, and 23.0%, respectively; HR of discontinuation for DPP4 inhibitors compared with metformin was 1.43 (95% CI 1.38–1.49, p < 0.001)
Ogundipe et al., 2021 [61]	Systematic review and meta-analysis	Proportion of days covered (PDC); medication possession ratio (MPR)	The number of days without a prescription gap of ≥ 90 days	Pooled analysis on 594,138 T2DM patients (aged 18 or older) identified in 34 studies	6 months, 1 year, 2 years, 3 year	The pooled estimate for PDC (and MPR) was 0.72 (95% CI 0.68 to 0.77, I^2^ = 99.5%); the pooled estimate for the percentage of adherent patients (PDC ≥ 0.8) was 56.9% (95% CI 49.3–64.4%, I^2^ = 99.9%); the pooled estimates for the percentages of persistent patients at six months, one year, two years, and three years were 75.6% (95% CI 71.5–79.5%, I^2^ = 99.8%), 60.0% (95% CI 57.0–62.0%, I^2^ = 99.8%), 52.8% (95% CI 51.6–59.8%, I^2^ = 99.8%), and 31.4% (95% CI 31.0–31.8%, I^2^ = 0%)
Kadowaki et al., 2018 [58]	The Medical Data Vision (MDV) database	N.A	The number of days without a prescription gap of ≥ 30 days	162,116 T2DM patients aged 40 or older with at least 1 prescription record of antidiabetic medication between January 1, 2014 and September 30, 2016	N.A	Besides metformin, treatment persistence was longest for DPP4 inhibitors (median 17.0 [95% CI 16.4–17.5] months); Persistence was longest with DPP4 inhibitors at all renal impairment stages (G1 to G4 +)
Ito et al., 2019 [60]	Prospective study by the Department of Diabetes, Metabolism and Kidney Disease, Edogawa Hospital (Tokyo, Japan)	The Diabetes Treatment Satisfaction Questionnaire (DTSQ)	N.A	79 T2DM patients aged 18 or older who switched treatment from daily DPP4 inhibitors to once-weekly trelagliptin between January 2017 to March 2018	3 months	The scores representing treatment satisfaction and medication adherence improved after switching from daily DPP-4 inhibitors to once-weekly trelagliptin (p < 0.01)
Gor et al., 2020 [57]	The MarketScan® commercial claims and encounters database	Proportion of days covered (PDC)	The number of days before the gap of two times the day’s supply or the end of follow-up	9,019 T2DM patients (aged 18 and above) with non-dialysis chronic kidney disease, who initiated either a DPP4 inhibitor or pioglitazone (7,002 DPP4 inhibitor users)	1 year	Mean one-year PDC for DPP4 inhibitors was 0.77 while mean PDC for pioglitazone was 0.72 (p < 0.01); the percentage of adherent patients (PDC ≥ 0.8) for DPP4 inhibitors was 59.5%, while that of pioglitazone was 52.4% (p < 0.01); OR of adherence for DPP inhibitor was 1.41 (95% CI 1.25–1.59, p < 0.01) compared with that for pioglitazone; 56.7% of patients on DPP4 were persistent compared with 46.3% of patients on pioglitazone (p < 0.01)
Nishimura et al., 2019 [27]	The Japan Medical Data Center (JMDC) database and the Medical Data Vision (MDV) database	Proportion of days covered (PDC)	Percentage of patients who remained on treatment after one-year follow-up	40,908 and 90,421 (from JMDC and MDV, respectively) adult T2DM patients aged 18 or older in Japan who had been issued at least one prescription for the index medication during January 2011 to December 2015	1 year	One-year persistence rates were 67.4% and 77.2% for treatment naïve patients identified from JMDC and MDV, respectively; One-year persistence rates were 73.5% and 78.8% for previously treated patients identified from JMDC and MDV; one-year adherence was 0.87 and 0.98 for treatment naive patients identified from JMDC and MDV; one-year adherence was 0.89 and 0.98 for previously treated patients identified from JMDC and MDV respectively
*Insulin*						
Wei et al., 2017 [63]	National pharmacy database from Walgreen Co. (Deerfield, IL)	N.A	Persistence was defined as remaining on the index medication during the 1-year follow-up period	247,102 T2DM patients aged ≥ 18 who filled ≥ 1 prescription for one of the index drugs along with ≥ 1 oral antidiabetic drug (OAD) between January 2013 and June 2013	1 year	66.8% of patients (95% confidence interval [CI] = 66.6 – 67.0) remained persistent after 1-year follow-up. The mean duration of persistence was 307.9 days (standard deviation [SD] = 85.2)
Bermeo-Cabrera et al., 2018 [64]	The diabetes clinic of a tertiary care center in Mexico City	Morisky-Green Questionnaire	N.A	200 T2DM patients on insulin treatment between March 2017 and December 2017	N.A	11% scored excellent adherence; 30.5% scored moderately good adherence; 58.5% scored poor adherence
Perez-Nieves et al., 2018 [65]	The Truven Health MarketScan® Research Databases	Proportion of days covered (PDC)	N.A	21,363 T2DM patients aged ≥ 18 who filled ≥ 1 prescription for the index drugs in 2012	3 years	33.8% of patients remained adherent (PDC after 3 years
Sambamoorthi et al., 2017 [62]	Medical, pharmacy, and laboratory claims data from Humana Medicare Advantage Prescription Drug (MAPD) plans	N.A	Persistence was defined as the absence of any 90-day gap between rapid-acting insulin prescriptions	3,927 elderly Medicare beneficiaries (65 years or older), who added therapy with rapid-acting insulin between July 2007 and December 2011	1 year	20.8% of patients remained persistent after 1-year follow-up
Hadjiyianni et al., 2017 [87]	Administrative claims database from Japan Medical Data Center	N.A	Persistence was defined as having a prescription gap of < 30 days	827 T2DM patients aged less than 70 years who were employed by middle-to-large size companies in Japan and with at least one pharmacy claim for the index drug between May 1, 2006 and April 30, 2013	1 year	36% of patients remained persistent. 42% had at least one gap of > 30-day prescription gap. 22% discontinued after the first prescription gap
*Glucagon-like peptide-1 receptor agonists*						
Mody et al., 2021 [75]	HealthCore Integrated Research Database (HIRD)	Proportion of days covered (PDC)	The number of days of continuous treatment without a gap in therapy of greater than 45 days	18,650 T2DM patients aged 18 or older who initiated one of the index medications between February 2018 and December 2018; matched cohorts through propensity score matching, consisting of 12,919 patients on dulaglutide, 3,852 patients on semaglutide, and 1,879 on exenatide BCise	6 months	Dulaglutide users had significantly higher mean PDC (0.75 vs. 0.67, p < 0.0001) and proportion of persistent patients (69.2% vs. 59.2%, p < 0.0001) compared with semaglutide users; dulaglutide users had significantly higher mean PDC (0.75 vs. 0.63, p < 0.0001) and proportion of persistent patients (67.9% vs. 50.6%, p < 0.0001) compared with exenatide BCise users
Tofe et al., 2021 [77]	Hospital recrods at Son Espases University Hospital, Palma de Mallorca, Spain	Days covered by medication fills as measured by quarterly evaluation of pharmacy claims	Discontinuation rates registered in patients' records at each visit during the follow-up period	298 T2DM patients aged 18 or older who initiated dulaglutide or sc semaglutide between January 2019 and June 2020; matched cohorts through propensity score matching consisting of 183 patients on dulaglutide and 115 patients on sc semaglutide	6 months	84.25% of dulaglutide users were adherent at 6 months; 83.71% of sc semaglutide users were adherent at 6 months; 20.5% of dulaglutide users discontinued at 6 months; 21.4% of sc semaglutide users discontinued at 6 months
Mody et al., 2018 [71]	HealthCore Integrated Research Database (HIRD)	Proportion of days covered (PDC)	The number of days of continuous treatment without a gap in therapy of greater than 45 days	1,970 T2DM patients aged 18 or older with at least one pharmacy claim for dulaglutide between November 1, 2014 and November 30, 2015	183 days	61% of patients were adherent (PDC ≥ 0.8); the mean PDC was 0.76; 37% patients discontinued; the mean number of days of persistent use of dulaglutide was 152 days
Uzoigwe et al., 2021 [74]	The Optum Clinformatics® Data Mart	Proportion of days covered (PDC)	The number of days without a prescription gap of > 60 days	56,715 T2DM patients aged 18 or older who initiated treatment with GLP-1RA agent between January 1, 2018 and April 30, 2019 (5.8% semaglutide QW, 49.2% dulaglutide, 30.3% liraglutide, 14.7% exenatide QW)	6 months or 1 year	The proportion of persistent patients at 6 months (1 year) was 74.0% (67.0%) for semaglutide QW; 66.4% (56.0%) for dulaglutide; 54.1% (40.4%) for liraglutide; 48.6% (35.5%) for exenatide QW; the proportion of adherent (PDC ≥ 0.8) patients at 6 months (1 year) was 44.7% (39.1%) for semaglutide QW; 53.8% (43.2%) for dulaglutide; 39.9% (30.0%) for liraglutide; 38.8% (27.7%) for exenatide QW
Durden et al., 2019 [73]	IBM Watson Health Explorys Universe Dataset	Proportion of days covered (PDC)	Continuous treatment lasting at least 12 or 18 months	8329 T2DM patients aged 18 or older who initiated GLP-1RA therapy between January 1, 2010 and January 1, 2017	1.5 year	The proportion of adherent (PDC ≥ 0.8) patients who experienced improvements in HbA1c level or body weight or both within 6 months was 45.0%, 43.4%, and 46.4%, respectively, compared with 37.1%, 39.0%, and 38.6% for those who did not (p < 0.001 for all comparisons); the proportion of discontinuation patients at 18 months who experienced improvements in HbA1c level or body weight or both within 6 months was 61.4%, 61.9%, and 60.0%, respectively, compared with 67.9%, 67.5%, and 66.7% for those who did not (p < 0.001 for all comparisons)
Rapuch et al., 2021 [88]	IQVIA Real World Data Adjudicated Pharmacy Claims	N.A	Continuous treatment without switching to a different medication class or without a prescription gap of at least twice the expected duration of the previous fill	15,074 T2DM patients aged 18 or older who initiated GLP-1RA therapy between January 1, 2015 and December 31, 2016	1 year	The proportion of persistent patients at 12 months was 39% for all GLP-1 RA agents; the median number of days of persistent treatment was 220 for all GLP-1 RA agents; the proportion of persistent patients at 12 months was 51% for dulaglutide; the proportion of persistent patients at 12 months was 35% for exenatide QW; the proportion of persistent patients at 12 months was 21% for exenatide BID; the proportion of persistent patients at 12 months was 36% for liraglutide
Alatorre et al., 2017 [76]	The Truven Health MarketScan Commercial Claims and Encounters, The Medicare Supplemental and Coordination of Benefits	Proportion of days covered (PDC)	The number of days of continuous treatment without > 60 days of gap in the prescription	16,197 T2DM patients aged 18 or older with at least 1 prescription claim for a GLP-1RA agent between November 5, 2014 to April 30, 2015	6 months	Mean PDC at 6 months was 0.72, 0.61, and 0.71 for dulaglutide, exenatide QW, and liraglutide, respectively; the proportion of patients who discontinued treatment before the end of 6-month follow-up was 26.2%, 48.4%, and 35.6% for dulaglutide, exenatide QW, and liraglutide, respectively
Svensson et al., 2021 [69]	The National Diabetes Register	N.A	Continuous treatment without ≥ 60 days prescription gap	17,361 T2DM patients aged 18 or older who initiated GLP-1RA treatment between May 23, 2015 and October 15, 2017	≥ 75 days	Proportion of persistent patients at one-year was 72.6% for exenatide QW, 80.9% for liraglutide, 71.2% for lixisenatide, and 87.7% for dulaglutide
Carls et al., 2017 [89]	The Optum/Humedica SmartFile Database	Proportion of days covered (PDC)	Continuous treatment without ≥ 30 days prescription gap	873 T2DM patients aged 18 or older who initiated DPP4 inhibitors or GLP1RA between January 2007 and December 2014 (221 GLP1RA users)	1 year	29% of patients were adherent (PDC ≥ 0.8), while 45.2% of GLP-1RA users discontinued
Nguyen et al., 2017 [72]	The Humana administrative claims database	Proportion of days covered (PDC)	N.A	5133 T2DM patients aged between 65 and 90, who initiated treatment with exenatide QW, exenatide BID, or liraglutide QD between January 1, 2020 and February 28, 2014	6 months	The proportion of adherent patients (PDC ≥ 0.8) at 6 month was 43.2%, 35.0%, 39.0% for exenatide QW, liraglutide, exenatide BID, respectively; the mean PDC at 6 months was 0.63, 0.57, 0.61 for exenatide QW, liraglutide, exenatide BID, respectively
*Thiazolidinediones*						
Flory et al., 2017 [7]	The OptumLabs Data Warehouse (OLDW)	Daily medication possession probability (daily MPP)	N.A	11,067 T2DM patients aged 18 or older who received a first electronic prescription for one of the index medications during the 2012 calendar year (409 pioglitazone users)	1 year	The daily MPP at one year was 0.36
McGovern et al., 2018 [1]	The Royal College of General Practitioners Research and Surveillance Centre (RCGP-RSC) database	N.A	Absence of a gap in prescriptions of greater than or equal to 90 days	60,327 T2DM patients who initiated treatment with one of the index medications between January 1, 2005 and December 31, 2015 (6,084 TZD users)	6 months, 1 year, 2 years, and 5 years	Median persistence was 1.55 years; percentages of persistent patients at 6 months, 1 year, 2 years, and 5 years were 75.6%, 61.2%, 43.0%, and 15.7%, respectively; HR of discontinuation for TZD compared with metformin was 1.71 (95% CI 1.64 to 1.77, p < 0.001)
Gor et al., 2020 [57]	The MarketScan® commercial claims and encounters database	Proportion of days covered (PDC)	The number of days before the gap of two times the days’ supply or the end of follow-up	9019 T2DM patients (aged 18 and above) with non-dialysis chronic kidney disease, who initiated either a DPP4 inhibitor or pioglitazone (2,017 pioglitazone users)	1 year	Mean PDC at one year was 0.72; the proportion of adherent (PDC ≥ 0.8) patients was 52.4%; the proportion of persistent patients at one year was 46.3%
Nishimura et al., 2019 [27]	The Japan Medical Data Center (JMDC) database and the Medical Data Vision (MDV) database	Proportion of days covered (PDC)	Percentage of patients who remained on treatment after one-year follow-up	40,908 and 90,421 (from JMDC and MDV, respectively) adult T2DM patients aged 18 or older in Japan who had been issued at least one prescription for the index medication during January 2011 to December 2015 (1,246 TZD users)	1 year	One-year persistence rates were 51.2% and 57.2% for treatment naïve patients identified from JMDC and MDV, respectively; One-year persistence rates were 50.0% and 48.1% for previously treated patients identified from JMDC and MDV; one-year adherence was 0.828 and 0.961 for treatment naive patients identified from JMDC and MDV; one-year adherence was 0.897 and 0.957 for previously treated patients identified from JMDC and MDV respectively

## Metformin

Metformin is well-tolerated and economic [21, 22], making it suitable for long-term treatment of T2DM. Furthermore, metformin has demonstrated the highest adherence and persistence rates in antidiabetic medications [2]. However, the adherence and persistence of metformin is still suboptimal [10, 23]. For example, the lowest daily medication possession probability (MPP) of metformin—i.e., the sum of days supplied by prescription fills during follow-up divided by the number of days in follow-up [24]—was only 0.46 [7]. Given that MPP ≥ 0.8 is generally accepted as the cut-off value for good adherence [25], the MPP value of 0.46 is certainly not optimal for a foundation medication like metformin. Likewise, the percentage of metformin users who continued treatment for one year ranged between 62.8 and 73.6% [26, 27]. The share of persistent metformin users declined to 48.5% and 27.7% at the end of second and fifth years of follow-up, respectively [26].

Baseline characteristics affect the adherence and persistence of metformin therapy. Higher baseline glycated hemoglobin (HbA1c) was associated with significantly lower rate of discontinuation. A percentage point increase in HbA1c was associated with 30% lower metformin persistence (95% confidence interval [95% CI]: 15–45%) [28]. Older patients were more persistent. For example, one year increase in age was associated with significantly better persistence (odds ratio or OR [95% CI]: 1.02 [1.02–1.02], p < 0.001) [29]. Using lower dose metformin (500 mg as opposed to 1000 mg) was associated with significantly lower discontinuation rate (OR [95% CI] of discontinuation of 500 mg metformin: 0.54 [0.37–0.76], p < 0.01) [28]. Similarly, taking fewer concomitant medication was associated with significantly better persistence (OR [95% CI]: 1.27 [1.20–1.33], p < 0.001) [29]. Patients using extended-release formulation were significantly more persistent than patients using immediate-release formulation (OR [95% CI] of persistence of extended-release formulation: 1.14 [1.10–1.18], p < 0.001) [30]. Table 2 summarizes the factors affecting the adherence and persistence of metformin therapy.

**Table 2:** Factors affecting adherence and persistence to metformin [19]

Patient-related barriers	Practitioner-related barriers	Treatment-related barriers
Difficulties in understanding the rationale for long-term metformin treatment	Poor health care team-patient relationship	Regimen complexity, especially in patients with multiple comorbid conditions
Difficulty swallowing; psychological difficulty swallowing tablets	Lack of times for in-depth communication with patient	Large size of or rough coating on metformin tablets
Memory problems (e.g., in older patients)	Lack of awareness of problems with treatment adherence	Inflexible treatment regimens
Socioeconomic factors (e.g., medication costs, lack of support during treatment)		Gastrointestinal side effects
Cultural attitudes and beliefs		

## Sulfonylureas

Sulfonylureas (including chlorpropamide, tolazamide, glipizide, glyburide, and glimepiride) are frequently prescribed to T2DM patients as second-line therapy despite potential hypoglycemic risks [20]. For sulfonylurea users, the proportion of days covered (PDC), or the number of days covered by prescription fills divided by the number of days between the first fill of the medication and the end of the measurement period [24], ranged from 0.62 and 0.72 [31, 32]. The percentage of patients who continued to take sulfonylureas at one year ranged between 50.4 and 68.9% [27, 31]. The longer the treatment period, the lower the persistence. For example, the percentage of patients who continued to take sulfonylureas declined to 51.3%, 47.1%, and 31.6% at the end of two, three and five years of follow-up, respectively [1, 32].

Sulfonylurea users were significantly less persistent than metformin users (hazard ratio or HR [95% CI] of discontinuation of sulfonylureas: 1.2 [1.16–1.24], p < 0.001) [1]. Moreover, the percentage of adherent patients (PDC ≥ 0.8) who used sulfonylurea as an add-on to metformin was significantly lower than that of patients who used sitagliptin as an add-on to metformin (55.9% versus 59.1%, p < 0.001) [32].

Sulfonylureas are commonly associated with hypoglycemia and weight gain [33, 34]. Approximately 20% of patients taking sulfonylureas may experience symptomatic hypoglycemia within six months after treatment initiation [35]. Moreover, sulfonylureas amplified weight gain in the first six months of treatment [36]. Such findings led to a speculation that the adverse events are the major reason for low adherence and persistence of sulfonylureas [33, 37]. However, no difference in treatment adherence was seen between patients who experienced hypoglycemic symptoms and those who did not [38]. Likewise, definitive evidence of the association between weight gain and poor adherence and persistence of sulfonylureas is still lacking.

## Sodium glucose co-transporter-2 inhibitors

Sodium glucose cotransporter-2 (SGLT2) inhibitors, e.g., canagliflozin, dapagliflozin, and empagliflozin, are generally well tolerated [39, 40]. The average PDC of SGLT2 inhibitors at one year was between 0.64 and 0.79 [41, 42]. Likewise, the proportion of patients who continued to take SGLT2 inhibitors at one year ranged between 44.3 and 72.1% [42, 43].

There was a difference in adherence and persistence among individual SGLT2 inhibitors. Canagliflozin was associated with significantly higher adherence and persistence than dapagliflozin (OR [95% CI] of adherence for canagliflozin compared with dapagliflozin: 1.29 [1.02–1.72]; HR [95% CI] of discontinuation of dapagliflozin: 1.28 [1.15–1.42]; both p < 0.001) [41]. Treatment with canagliflozin is suspected to be associated with increased risks of lower extremity amputation and skeletal fractures, besides other adverse events commonly ascribed to SGLT2 inhibitors [44]. However, the impact of these adverse effects on the adherence and persistence of canagliflozin has not been fully investigated, warranting further studies. Similarly, empagliflozin users were significantly more adherent and persistent than dapagliflozin users (OR [95% CI] of adherence and persistence of empagliflozin: 1.39 [1.29–1.51] and 1.14 [1.06–1.22], respectively; both p < 0.01) [42]. No study has directly compared the adherence and persistence rates between canagliflozin and empagliflozin.

The persistence rate of SGLT2 inhibitors was comparable to, and in some case even higher than, well-persisted antidiabetic medication classes (i.e., metformin and dipeptidyl peptidase-4 inhibitors). For example, SGLT2 inhibitor users were as persistent as metformin users (HR [95% CI] of discontinuation of SGLT2 inhibitors: 1.04 [0.93–1.17], p = 0.458) [2]. Likewise, over 50% of SGLT2 inhibitor users and metformin users remained persistent into the second year of treatment, while the majority of patients treated with other medication classes discontinued [2]. Furthermore, the discontinuation rate (i.e., having a prescription gap of > 90 days) of canagliflozin users was 12% less (p < 0.001) than that of sitagliptin (a dipeptidyl peptidase-4 inhibitor) users [45]

SGLT2 inhibitors block glucose reabsorption in the renal proximal tubules of the kidneys [41, 46, 47]. Their distinct mechanism of action entails a distinct set of adverse events, such as orthostatic hypotension, ketoacidosis, and most notably, genitourinary tract infections [48, 49]. The unique safety profile of SGLT2 inhibitors led to a distinct factor for persistence. For example, higher estimated glomerular filtration rate (eGFR) was associated with significantly lower persistence rate (the beta coefficient [standard error] for discontinuation per one-unit increase in eGFR: 0.01 [± 0.00], p < 0.001) [49]. The increased likelihood of genitourinary tract infections due to the hyperfiltration of urinary glucose excretion was proposed as a reason for the association [49]. Similar conclusions were made by other studies [48, 50].

On the other hand, the factors for persistence commonly observed in other antidiabetic medications also applied to SGLT2 inhibitors. For example, younger age [41], female gender [41, 49], higher baseline anxiety [41], higher baseline HbA1c [49], and baseline insulin use [41, 51] were associated with significantly higher discontinuation rates in SGLT2 inhibitor users. In contrast, lower starting dose [41], perceived feeling of improved clinical outcome [41, 51], and taking fewer number of concomitant medications [49, 51] were associated with significantly lower discontinuation rates in SGLT2 inhibitor users.

## Dipeptidyl peptidase-4 inhibitors

As of July 2021, four dipeptidyl peptidase-4 (DPP4) inhibitors (sitagliptin, saxagliptin, linagliptin, and alogliptin) are in clinical use in the US [52]. Other DPP4 inhibitors that are being prescribed worldwide include anagliptin, tenegliptin, vildagliptin, omarigliptin, and trelagliptin [53]. DPP4 inhibitors prevent the degradation of glucagon like peptide-1 (GLP1) and stimulate postprandial insulin secretion in a glucose-dependent manner [54]. DPP4 inhibitors are generally well tolerated and pose little risk of hypoglycemia and weight gain [55, 56]. This safety profile leads to good adherence and persistence in DPP4 inhibitor users. The mean PDC at one year of DPP4 inhibitors ranged from 0.67 to 0.77 [56, 57]. The percentage of patients who remained persistent to DPP4 inhibitors at one year was between 56.7 and 78.8% [27, 57]. The median persistence of DPP4 inhibitors was approximately seventeen months [2, 58].

A comparison between individual DPP4 inhibitors showed that sitagliptin and saxagliptin demonstrated similar adherence and persistence rates [56]. On the other hand, sitagliptin and saxagliptin were associated with significantly better adherence and persistence than linagliptin (OR [95% CI] of adherence for sitagliptin and saxagliptin: 1.40 [1.25–1.57] and 1.46 [1.29–1.66], respectively; HR [95% CI] of discontinuation for sitagliptin and saxagliptin: 0.88 [0.82–0.94] and 0.85 [0.79–0.91], respectively; all p < 0.001) [56].

The persistence of once-daily (QD) DPP4 inhibitors was comparable to that of twice-daily (BID) DPP4 inhibitors (HR [95% CI] of discontinuation for BID regimen: 1.022 [0.994–1.050], p = 0.1187) [59]. Similarly, the difference between the adherence rates of BID regimen and QD regimen was not significant (OR [95% CI] of adherence for BID regimen: 0.945 [0.780–1.145], p = 0.5636) [59]. On the other hand, QD regimen showed significantly higher adherence and persistence rates than once-weekly (QW) regimen (HR [95% CI] of discontinuation for QW regimens: 1.699 [1.585–1.822], p < 0.0001; OR [95% CI] of adherence for QW regimen: 0.029 [0.024–0.036], p < 0.0001) [59]. However, there are also conflicting results. In a prospective study, adherence was improved by 0.1 point (p = 0.03) on the Diabetes Treatment Satisfactions Questionnaire (DTSQ) scale after patients switched from QD DPP4 inhibitors (i.e., sitagliptin, vildagliptin, alogliptin, linagliptin, and teneligliptin) to QW trelagliptin [60].

The persistence of DPP4 inhibitors is lower than that of metformin (HR [95% CI] of discontinuation for DPP4 inhibitors: 1.43 [1.38–1.49], p < 0.001) [2]. However, DPP4 inhibitors are well adhered and persisted in T2DM patients with impaired kidney functions at all renal impairment stages [58]. Similarly, the adherence of DPP4 inhibitors in patients with chronic kidney disease (CKD) was significantly higher (OR [95% CI] of adherence for DPP4 inhibitors: 1.41 [1.25–1.59], p < 0.01) than pioglitazone, a well-established second-line therapy for T2DM patients with CKD [57].

The speculation that regimen complexity or the severity of diabetes may not necessarily lower adherence to DPP4 inhibitors [23] was supported by several studies [57–59]. Common reasons for discontinuing DPP4 inhibitors were inadequate glycemic control and intolerance, as with other antidiabetic medications [61].

## Insulin

Insulin is key to improving the glycemic outcome in many T2DM patients. However, the adherence and persistence rates of insulin treatment are generally suboptimal. For example, one-year persistence rates of insulin treatment, measured as the percentage of patients who remained on therapy, were only 20–66.8% [62, 63]. Similarly, adherence to insulin was low; 58.5% of T2DM patients on insulin therapy scored poor adherence (scoring below 6) on the Morisky-Green Questionnaire [64]. The adherence to insulin treatment was inversely related to treatment period. The proportion of adherent patients (i.e., those with PDC ≥ 0.8) on basal insulins dropped to 33.8% three years after treatment initiation [65]. Generally, basal insulins demonstrated better persistence than rapid-acting and short-acting insulins [2]. The major findings of previous studies that analyzed the adherence and persistence to insulin therapy are summarized in Table 1.

Simple reminding has been insufficient to improve the adherence and persistence of insulin treatment. In a recent randomized clinical trial, individualized interventions such as quarterly educational mailings, telephone consultation by pharmacists, and text reminders did not improve insulin persistence despite increasing intensity of the interventions [66]. Multifaceted approaches are crucial to adequately address insulin non-adherence and non-persistence. Table 3 summarizes the factors affecting adherence and persistence to insulin therapy.

**Table 3:** Factors affecting adherence and persistence to insulin therapy

Factors that improve adherence and persistence	Factors that lower adherence and persistence
Using analog insulin rather than human or animal-derived insulin [90]	Having high baseline hypoglycemia or fear of hypoglycemia [64, 90–92]
Using basal insulin rather than rapid- or short-acting insulin [2]	Having high comorbidity burden [90]
Being in age group (40–69) [90]	Experiencing or having fear of weight gain [92, 93]
Experiencing improved glycemic control [93]	Experiencing or having fear of pain from injection [64, 93]
Having access to support system formed primarily of physicians and healthcare professionals [93]	Feeling financially burdened [64, 94]
Using injection pen rather than vial or syringe [63]	Having complex regimen [64]
Having been treated for diabetes for longer duration [64]	Starting with higher dose [64]
Having higher baseline HbA1c level [90, 92]	Feeling that insulin treatment interferes with daily activities [64]

## Glucagon-Like peptide-1 receptor agonists

Glucagon-like peptide-1 receptor agonists (GLP-1RA) improve glycemic control and cardiovascular factors, reduce body weight, and rarely induce hypoglycemia [67]. GLP-1RA agents are preferred second-line treatment options for T2DM patients with cardiovascular comorbidities [68]. Furthermore, GLP-1RA agents are recommended as the first injectable medication before insulin [69]. As of 2021, nine formulations of injectable GLP-1RA agents have been approved worldwide (Table 4). Oral semaglutide (brand name: Rybelsus®) was the first oral formulation of GLP-1RA approved by the US Food and Drug Administration for the treatment of T2DM [70].

**Table 4:** List of injectable GLP-1RA agents currently in clinical use worldwide

Drug	Dosing frequency
Exenatide	Twice a day (BID)
Liraglutide	Once a day (QD)
Exenatide	Once a week (QW)
Albiglutide	Once a week (QW)
Dulaglutide prefilled pen	Once a week (QW)
Exenatide pen	Once a week (QW)
Lixisenatide	Once a day (QD)
Exenatide auto-injector	Once a week (QW)
Semaglutide	Once a week (QW)

The average PDC of injectable GLP-1RA agents at six months ranged from 0.61 to 0.76 [71, 72]. The proportion of patients who continued treatment with injectable GLP-1RA at six months was between 32.1 and 74.0% [73, 74]. The adherence and persistence of oral semaglutide remain to be seen.

Injectable GLP-1RA agents differ in dosing regimens, need for dose titration and reconstitution, and administration device features [75]. These differences led to differences in adherence and persistence rates among individual GLP-1RA agents. Dulaglutide showed significantly higher persistence than other GLP-1RA agents (HR [95% CI] of discontinuation compared with dulaglutide: 2.5 [2.1–3.0] for exenatide QW, 1.6 [1.5–1.8] for liraglutide, 1.4 [1.3–1.5] for semaglutide, and 2.8 [2.3–3.3] for lixisenatide; all p < 0.001) [69, 75]. Similarly, dulaglutide was associated with significantly higher adherence than other GLP-1RA agents (OR [95% CI] of adherence compared with dulaglutide: 0.63 [0.55–0.73] for albiglutide, 0.32 [0.28–0.37] for exenatide BID, 0.48 [0.43–0.53] for exenatide QW, and 0.65 [0.59–0.71] for liraglutide; all p < 0.05) [76].

In general, GLP-1RA agents with QW regimen demonstrated significantly better adherence and persistence than GLP-1RA agents with QD or BID regimen [67, 72, 74, 76]. In terms of delivery method, GLP-1RA agents using simple delivery systems (single-use pen or auto-injector device) had significantly higher adherence and persistence than GLP-1RA using multi-use pen or syringe [67, 71, 75–77]. Furthermore, experiencing early response (defined as improvements in HbA1c and body weight within six months after treatment initiation) was associated with significantly higher adherence and persistence in GLP-1RA users [73]. Other factors for the adherence and persistence of GLP-1RA agents are summarized in Table 5.

**Table 5:** Factors affecting the adherence and persistence to GLP-1RA

Reasons for treatment discontinuation	Factors for higher adherence and persistence
Inadequate blood glucose control [67]	Initiating treatment with low dose [67, 75]
Gastrointestinal side effects (including nausea/vomiting) [67, 75, 77]	Ease of use of injection device [75, 77]
Preference for oral medication over injection [95]	Weekly dosing rather than daily or twice daily dosing [72]
High cost [95]	Early (within 6 months) weight loss [73]
Injection site reaction [95]	Early (within 6 months) HbA1c level reduction [73]
Inadequate body weight reduction [73]	
Inconvenience of injection schedule [67, 71]	
Injection-related concerns (including pain and fear) [76]	

## Thiazolidinediones

Thiazolidinediones (TZDs), including pioglitazone, are agonists of peroxisome proliferator-activated receptor-γ (PPAR-γ) used in the treatment of T2DM. TZDs reduce plasma glucose by directly activating PPAR-γ and improving insulin sensitivity [78, 79]. Furthermore, TZDs, along with DPP4 inhibitors, are the major treatment option for T2DM patients with impaired renal functions [57]. Despite their therapeutic benefits, few studies published since 2017 analyzed the adherence and persistence of TZDs due partly to the diminished market share (in case of rosiglitazone) or withdrawal from the market (in case of troglitazone) [20, 80–82]. Still, the adherence of TZDs that are still being prescribed (e.g., pioglitazone) has been reported to range from 0.36 (measured in the daily MPP) to 0.72 (measured in PDC) [7, 57]. The proportion of patients who remained persistent with TZDs for one-year varied from 46.3 to 75.6% (Table 1) [1, 57].

The safety issues surrounding TZDs significantly lowered adherence and persistence. For example, after the FDA issued a safety warning in June 2011 for pioglitazone and its possible link to bladder cancer, the discontinuation rate of pioglitazone increased significantly from 36.3% in the year 2010 to 41.0% in the year 2011 (p < 0.01) [57].

## Conclusion

This article provided a comprehensive review of the adherence and persistence of major antidiabetic medications, i.e., metformin, sulfonylureas, SGLT2 inhibitors, DPP4 inhibitors, insulins, GLP-1RA agents, and TZDs. The adherence and persistence of major antidiabetic medications were not optimal, given that PDC ≥ 0.8 and the proportion of persistent patients ≥ 80% are generally recognized as optimum [25].

Most studies reported adherence in PDC and defined patients with PDC ≥ 0.8 as adherent. Persistence was predominantly defined as continuous treatment without a prescription gap of more than 90 days. In most studies, the proportion of patients who remained persistent was used as the measure of the drug’s persistence rate.

The highest rate of adherence and persistence was consistently observed in metformin users. Second to metformin were SGLT2 inhibitors. Injectable therapies such as insulin and GLP-1RA agents trailed low on the adherence and persistence rates. To the best of our knowledge, no studies published since the year 2017 analyzed the adherence and persistence of TZDs independently.

Most studies pointed out that the prevalence and severity of adverse events is associated with low medication adherence and persistence. Baseline characteristics (e.g., baseline HbA1c level), socioeconomic factors (e.g., medication costs and insurance status), demographic information (e.g., age, gender, or ethnicity), and comorbidity profiles also had significant impacts on adherence and persistence in T2DM patients.

It is important to note that reports on the adherence and persistence rates varied, depending on study design, data source, and patient sample. Using different definitions of adherence (e.g., daily MPP or PDC) and of persistence (e.g., continuous treatment without a prescription gap of over 60 days or 90 days) may have also contributed to the variations in the findings. In the similar vein, primary adherence (i.e., the rate at which patients fill prescriptions for the first time after treatment initiation) is critical for timely treatment of both acute and chronic conditions [83]. Despite its clinical significance, we found that the primary adherence of antidiabetic medications has not been extensively covered in the literature. Moreover, most studies measured adherence and persistence over one year after the initiation of antidiabetic drugs. Because these measures are inversely related to the duration of follow-up, using different observation periods may have led to different results. Lastly, most studies relied on electronic health records (EHR) and claims data to analyze the adherence and persistence. Thus, it should be acknowledged that a purely claims-based study may have underestimated adherence and persistence by leaving out the patients who paid out of pocket. On the contrary, a purely EHR-based study may have overestimated the adherence and persistence because there are significantly more provider attempts to prescribe a drug than there are patients voluntarily taking the drug in the long term [7].

This review article aimed to address the following points. First, this review article offers a concise summary of the adherence and persistence rates of antidiabetic medications that comprise of the most of the proportional market share worldwide. Secondly, the findings summarized in this article may help guide the clinicians and the patients to make informed treatment decisions. Study results from randomized clinical trials confirm the effectiveness and safety of a medication. However, the results may not always translate into everyday clinical benefit, as there are diverse factors at work, including adherence and persistence. In this light, knowing the treatment-related factors and patient-level information affecting the behavior of medication (e.g., treatment adherence and persistence) use may equip the medical community to identify the beneficiaries of the drug’s effectiveness with higher granularity. Lastly, information contained in this article may provide insight into the paths for the development of new antidiabetic medications or injection devices that are more amenable to fostering adherence and persistence.

Because the treatment of diabetes relies heavily on antidiabetic medications, along with lifestyle modification, dietary interventions, and other medications for comorbid conditions, ensuring adherence and persistence is key to adequately managing the disease. Moreover, adherence and persistence are clinically important phenomena with implications for research and clinical practice. Therefore, clinicians should be aware of the challenges concerning adherence and persistence to antidiabetic medications. It is imperative that they pay attention to how the multifactorial nature of medication non-use undermines patients’ quality of life and clinical outcomes.

## Data Availability

Data sharing is not applicable to this article as no datasets were generated or analyzed during the current study.

## Abbreviations

HbA1c: Glycated hemoglobin
T2DM: Type 2 diabetes mellitus
MPP: Medication possession probability
OR: Odds ratio
95% CI: 95% Confidence interval
PDC: Proportion of days covered
HR: Hazard ratio
SGLT2: Sodium glucose cotrasporter-2
eGFR: Estimated glomerular filtration rate
DPP4: Dipeptidyl peptidase-4
GLP1: Glucagon like peptide-1
QD: Once-daily
BID: Twice-daily
QW: Once-weekly
DTSQ: Diabetes Treatment Satisfaction Questionnaire
CKD: Chronic kidney disease
TZD: Thiazolidinediones
PPAR-γ: Peroxisome proliferator-activated receptor-γ
EHR: Electronic health records

## References

[1] McGovern A (2018). A class comparison of medication persistence in people with type 2 diabetes: a retrospective observational study. Diabetes Therapy.

[2] McGovern A (2018). Comparison of medication adherence and persistence in type 2 diabetes: a systematic review and meta-analysis. Diabetes Obes Metab.

[3] Jermendy G (2012). Persistence of initial oral antidiabetic treatment in patients with type 2 diabetes mellitus. Med Sci Monit Int Med J Exp Clin Res.

[4] Organization WH. Adherence to long-term therapies: evidence for action. World Health Organization; 2003.

[5] Abegunde DO (2007). The burden and costs of chronic diseases in low-income and middle-income countries. Lancet.

[6] Asche C, LaFleur J, Conner C (2011). A review of diabetes treatment adherence and the association with clinical and economic outcomes. Clin Ther.

[7] Flory J (2017). Comparative adherence to diabetes drugs: an analysis of electronic health records and claims data. Diabetes Obes Metab.

[8] Jha AK (2012). Greater adherence to diabetes drugs is linked to less hospital use and could save nearly $5 billion annually. Health Aff.

[9] Kennedy-Martin T, Boye KS, Peng X (2017). Cost of medication adherence and persistence in type 2 diabetes mellitus: a literature review. Patient Prefer Adherence.

[10] Iglay K (2015). Meta-analysis of studies examining medication adherence, persistence, and discontinuation of oral antihyperglycemic agents in type 2 diabetes. Curr Med Res Opin.

[11] Baser O (2013). Real-world outcomes of initiating insulin glargine-based treatment versus premixed analog insulins among US patients with type 2 diabetes failing oral antidiabetic drugs. ClinicoEcon Outcomes Res CEOR.

[12] Aikens JE, Piette JD (2013). Longitudinal association between medication adherence and glycaemic control in type 2 diabetes. Diabet Med.

[13] Rozenfeld Y (2008). Oral antidiabetic medication adherence and glycemic control in managed care. Am J Managed Care.

[14] Hong JS, Kang HC (2011). Relationship between oral antihyperglycemic medication adherence and hospitalization, mortality, and healthcare costs in adult ambulatory care patients with type 2 diabetes in South Korea. Med Care.

[15] Currie CJ (2012). The impact of treatment noncompliance on mortality in people with type 2 diabetes. Diabetes Care.

[16] Egede LE (2012). Medication nonadherence in diabetes: longitudinal effects on costs and potential cost savings from improvement. Diabetes Care.

[17] Sabaté E, Sabaté E. Adherence to long-term therapies: evidence for action. World Health Organization; 2003.

[18] Chandran A (2015). Adherence to insulin pen therapy is associated with reduction in healthcare costs among patients with type 2 diabetes mellitus. Am Health Drug Benefits.

[19] Christofides EA (2019). Practical insights into improving adherence to metformin therapy in patients with type 2 diabetes. Clin Diabetes.

[20] Montvida O (2018). Long-term trends in antidiabetes drug usage in the US: real-world evidence in patients newly diagnosed with type 2 diabetes. Diabetes Care.

[21] Association AD (2019). Summary of revisions: standards of medical care in diabetes—2019. Diabetes Care.

[22] Maruthur NM (2016). Diabetes medications as monotherapy or metformin-based combination therapy for type 2 diabetes: a systematic review and meta-analysis. Ann Intern Med.

[23] Cramer JA (2004). A systematic review of adherence with medications for diabetes. Diabetes Care.

[24] Nau DP (2012). Proportion of days covered (PDC) as a preferred method of measuring medication adherence.

[25] Karve S (2009). Good and poor adherence: optimal cut-point for adherence measures using administrative claims data. Curr Med Res Opin.

[26] Horsburgh S (2021). Patterns of metformin monotherapy discontinuation and reinitiation in people with type 2 diabetes mellitus in New Zealand. PLoS ONE.

[27] Nishimura R (2019). Treatment patterns, persistence and adherence rates in patients with type 2 diabetes mellitus in Japan: a claims-based cohort study. BMJ Open.

[28] Flory JH (2018). Identifying prevalence and risk factors for metformin non-persistence: a retrospective cohort study using an electronic health record. BMJ Open.

[29] Shani M, Lustman A, Vinker S (2017). Diabetes medication persistence, different medications have different persistence rates. Prim Care Diabetes.

[30] Flory JH, Mushlin AI (2020). Effect of cost and formulation on persistence and adherence to initial metformin therapy for type 2 diabetes. Diabetes Care.

[31] Bell KF (2017). Comparing medication adherence and persistence among patients with type 2 diabetes using sodium-glucose cotransporter 2 inhibitors or sulfonylureas. Am Health Drug Benefits.

[32] Bloomgarden ZT (2017). Adherence, persistence, and treatment discontinuation with sitagliptin compared with sulfonylureas as add-ons to metformin: a retrospective cohort database study: 在二甲双胍治疗的基础上比较加用西格列汀治疗与加用磺脲类药物治疗的依从性, 持久性以及治疗中止: 一项回顾性队列数据库研究. J Diabetes.

[33] Nunes AP (2017). Hypoglycaemia seriousness and weight gain as determinants of cardiovascular disease outcomes among sulfonylurea users. Diabetes Obes Metab.

[34] Thulé PM, Umpierrez G (2014). Sulfonylureas: a new look at old therapy. Curr Diabetes Rep.

[35] Jennings AM, Wilson RM, Ward JD (1989). Symptomatic hypoglycemia in NIDDM patients treated with oral hypoglycemic agents. Diabetes Care.

[36] Carls G (2017). Real-world weight change among patients treated with glucagon-like peptide-1 receptor agonist, dipeptidyl peptidase-4 inhibitor and sulfonylureas for type 2 diabetes and the influence of medication adherence. Obes Sci Pract.

[37] Pi-Sunyer FX (2009). The impact of weight gain on motivation, compliance, and metabolic control in patients with type 2 diabetes mellitus. Postgrad Med.

[38] Popoviciu S, Alionescu A, Sisic I (2019). Prevalence of hypoglycemia, treatment satisfaction, adherence and their associations with glycemic goals in patients with type 2 diabetes mellitus treated with sulfonylureas: findings from the Real-Life Effectiveness and Care Patterns of Diabetes Management (RECAP-DM) in Romania. Romanian J Diabetes Nutr Metab Dis.

[39] Khunti N, Khunti N, Khunti K (2019). Adherence to type 2 diabetes management. Br J Diabetes.

[40] Hirsch IB, Molitch ME (2013). Clinical decisions. Glycemic management in a patient with type 2 diabetes. N Engl J Med.

[41] Cai J, Divino V, Burudpakdee C (2017). Adherence and persistence in patients with type 2 diabetes mellitus newly initiating canagliflozin, dapagliflozin, DPP-4s, or GLP-1s in the United States. Curr Med Res Opin.

[42] Ofori-Asenso R (2019). Adherence, persistence, and switching among people prescribed sodium glucose co-transporter 2 inhibitors: a nationwide retrospective cohort study. Adv Therapy.

[43] Ito Y (2019). Real-world effectiveness of sodium glucose co-transporter-2 inhibitors in Japanese patients with diabetes mellitus. Diabetes Therapy.

[44] Neal B (2017). Canagliflozin and cardiovascular and renal events in type 2 diabetes. N Engl J Med.

[45] Thayer S (2017). HbA1c outcomes in patients treated with canagliflozin versus sitagliptin in US health plans. Clin Ther.

[46] Chao EC, Henry RR (2010). SGLT2 inhibition—a novel strategy for diabetes treatment. Nat Rev Drug Discov.

[47] Jabbour S, Goldstein B (2008). Sodium glucose co-transporter 2 inhibitors: blocking renal tubular reabsorption of glucose to improve glycaemic control in patients with diabetes. Int J Clin Pract.

[48] Tumminia A (2021). Efficacy, renal safety and tolerability of sodium-glucose cotransporter 2 inhibitors (SGLT2i) in elderly patients with type 2 diabetes: a real-world experience. Prim Care Diabetes.

[49] Fadini G (2020). Predictors of early discontinuation of dapagliflozin versus other glucose-lowering medications: a retrospective multicenter real-world study. J Endocrinol Invest.

[50] Lorenzo MG et al. 4CPS-013 Discontinuation of sodium-glucose co-transporter 2 inhibitors due to recurrent genitourinary infections. British Medical Journal Publishing Group. 2018.

[51] Buysman EK (2017). Retrospective study on the impact of adherence in achieving glycemic goals in type 2 diabetes mellitus patients receiving canagliflozin. Adv Therapy.

[52] Srinivasa Venkata Siva Kumar Kasina KMB. Dipeptidyl Peptidase IV (DPP IV) inhibitors. StatPerals Publishing, Treasure Island (FL); 2021. 31194471

[53] Gallwitz B (2019). Clinical use of DPP-4 inhibitors. Front Endocrinol.

[54] Bhavadasan K, Davis AM, Kolanthavel B (2019). Impact of dipeptidyl peptidase-4 inhibitors on glycemic control and cardiovascular safety with adherence: an overview. Dubai Diabetes Endocrinol J.

[55] Dicker D (2011). DPP-4 inhibitors: impact on glycemic control and cardiovascular risk factors. Diabetes Care.

[56] Rascati KL (2017). Adherence, persistence, and health care costs for patients receiving dipeptidyl peptidase-4 inhibitors. J Manag Care Spec Pharm.

[57] Gor D (2020). Adherence and persistence with DPP-4 inhibitors versus pioglitazone in type 2 diabetes patients with chronic kidney disease: a retrospective claims database analysis. J Manag Care Spec Pharm.

[58] Kadowaki T (2018). Persistence of oral antidiabetic treatment for type 2 diabetes characterized by drug class, patient characteristics and severity of renal impairment: a Japanese database analysis. Diabetes Obes Metab.

[59] Oh A (2020). Comparison of persistence and adherence between DPP-4 inhibitor administration frequencies in patients with type 2 diabetes mellitus in Japan: a claims-based cohort study. Curr Med Res Opin.

[60] Ito H (2019). Changes in medication adherence and unused drugs after switching from daily dipeptidyl peptidase-4 inhibitors to once-weekly trelagliptin in patients with type 2 diabetes. Diabetes Res Clin Pract.

[61] Ogundipe O (2021). Real-world adherence, persistence, and in-class switching during use of dipeptidyl peptidase-4 inhibitors: a systematic review and meta-analysis involving 594,138 patients with type 2 diabetes. Acta Diabetol.

[62] Sambamoorthi U (2017). Persistence with rapid-acting insulin and its association with A1C level and severe hypoglycemia among elderly patients with type 2 diabetes. Curr Med Res Opin.

[63] Wei W (2017). Benchmarking insulin treatment persistence among patients with type 2 diabetes across different US payer segments. J Manag Care Spec Pharm.

[64] Bermeo-Cabrera J (2018). Insulin adherence in type 2 diabetes in Mexico: behaviors and barriers. J Diabetes Res.

[65] Perez-Nieves M (2018). Adherence to basal insulin therapy among people with type 2 diabetes: a retrospective cohort study of costs and patient outcomes. Diabetes Therapy.

[66] Lauffenburger J (2018). Effect of targeted insulin adherence interventions for glycemic control with predictive analytics: the TARGIT-diabetes randomized clinical trial. Circulation.

[67] Guerci B (2019). Efficacy and adherence of glucagon-like peptide-1 receptor agonist treatment in patients with type 2 diabetes mellitus in real-life settings. Diabetes Metab.

[68] Association AD (2020). 9. Pharmacologic approaches to glycemic treatment: standards of medical care in diabetes—2020. Diabetes Care.

[69] Svensson AM (2021). Treatment persistence in patients with type 2 diabetes treated with glucagon-like peptide-1 receptor agonists in clinical practice in Sweden. Diabetes Obes Metab.

[70] Nordisk N. Rybelsus (semaglutide) tablets [prescribing information]. Plainsboro, NJ, Novo Nordisk, 2019.

[71] Mody R (2018). Real-world effectiveness, adherence and persistence among patients with type 2 diabetes mellitus initiating dulaglutide treatment. Curr Med Res Opin.

[72] Nguyen H, Dufour R, Caldwell-Tarr A (2017). Glucagon-like peptide-1 receptor agonist (GLP-1RA) therapy adherence for patients with type 2 diabetes in a medicare population. Adv Therapy.

[73] Durden E (2019). The effect of early response to GLP-1 RA therapy on long-term adherence and persistence among type 2 diabetes patients in the United States. J Manag Care Spec Pharm.

[74] Uzoigwe C (2021). Semaglutide once-weekly persistence and adherence versus other GLP-1 RAs in patients with type 2 diabetes in a US real-world setting. Diabetes Therapy.

[75] Mody R (2021). Adherence and persistence among patients with type 2 diabetes initiating dulaglutide compared with semaglutide and exenatide BCise: 6-month follow-up from US real-world data. Diabetes Obes Metab.

[76] Alatorre C (2017). Treatment patterns in patients with type 2 diabetes mellitus treated with glucagon-like peptide-1 receptor agonists: higher adherence and persistence with dulaglutide compared with once-weekly exenatide and liraglutide. Diabetes Obes Metab.

[77] Tofé S (2021). An observational study evaluating effectiveness and therapeutic adherence in patients with Type 2 diabetes initiating dulaglutide vs subcutaneous semaglutide in Spain. Endocr Metab Sci.

[78] Kemnitz JW (1994). Pioglitazone increases insulin sensitivity, reduces blood glucose, insulin, and lipid levels, and lowers blood pressure, in obese, insulin-resistant rhesus monkeys. Diabetes.

[79] Lebovitz HE (2001). Rosiglitazone monotherapy is effective in patients with type 2 diabetes. J Clin Endocrinol Metab.

[80] Cheng D, Gao H, Li W (2018). Long-term risk of rosiglitazone on cardiovascular events—a systematic review and meta-analysis. Endokrynol Pol.

[81] Davidson MA (2018). Thiazolidinedione drugs in the treatment of type 2 diabetes mellitus: past, present and future. Crit Rev Toxicol.

[82] Tang H (2018). Pioglitazone and bladder cancer risk: a systematic review and meta-analysis. Cancer Med.

[83] Fischer MA (2010). Primary medication non-adherence: analysis of 195,930 electronic prescriptions. J Gen Intern Med.

[84] Naffaa M (2020). Adherence to metformin and the onset of rheumatoid arthritis: a population-based cohort study. Scand J Rheumatol.

[85] Walker EA (2020). Long-term metformin adherence in the diabetes prevention program outcomes study. BMJ Open Diabetes Res Care.

[86] Moura CS (2018). Treatment discontinuation and clinical events in type 2 diabetes patients treated with dipeptidyl peptidase-4 inhibitors or NPH insulin as third-line therapy. J Diabetes Res.

[87] Hadjiyianni I (2017). Basal insulin persistence, associated factors, and outcomes after treatment initiation: a retrospective database study among people with type 2 diabetes mellitus in Japan. Diabetes Therapy.

[88] Rapuch SZ (2021). Treatment patterns and persistence with GLP-1 RA treatments among patients with type 2 diabetes in France: a retrospective cohort analysis. Diabetes Therapy.

[89] Carls GS (2017). Understanding the gap between efficacy in randomized controlled trials and effectiveness in real-world use of GLP-1 RA and DPP-4 therapies in patients with type 2 diabetes. Diabetes Care.

[90] He X (2017). Insulin adherence and persistence among Chinese patients with type 2 diabetes: a retrospective database analysis. Patient Prefer Adherence.

[91] Li P (2019). Early hypoglycaemia and adherence after basal insulin initiation in a nationally representative sample of medicare beneficiaries with type 2 diabetes. Diabetes Obes Metab.

[92] Perez-Nieves M (2017). Basal insulin initiation use and experience among people with type 2 diabetes mellitus with different patterns of persistence: results from a multi-national survey. Curr Med Res Opin.

[93] Idris I (2019). Associated factors that influenced persistence with basal analog insulin therapy among people with type 2 diabetes: an exploratory analysis from a UK real-world sample. Prim Care Diabetes.

[94] Davies M (2013). Real-world factors affecting adherence to insulin therapy in patients with Type 1 or Type 2 diabetes mellitus: a systematic review. Diabet Med.

[95] Sikirica MV (2017). Reasons for discontinuation of GLP1 receptor agonists: data from a real-world cross-sectional survey of physicians and their patients with type 2 diabetes. Diabetes Metab Syndrome Obes Targets Therapy.

